# The Adenovirus Genome Contributes to the Structural Stability of the Virion

**DOI:** 10.3390/v6093563

**Published:** 2014-09-24

**Authors:** Bratati Saha, Carmen M. Wong, Robin J. Parks

**Affiliations:** 1 Regenerative Medicine Program, Ottawa Hospital Research Institute, Ottawa, Ontario K1H 8L6, Canada; E-Mails: bsaha@ohri.ca (B.S.); cawong@ohri.ca (C.M.W.); 2 Department of Biochemistry, Microbiology and Immunology, University of Ottawa, Ottawa, Ontario K1H 8M5, Canada; 3 Centre for Neuromuscular Disease, University of Ottawa, Ottawa, Ontario K1H 8M5, Canada; 4 Department of Medicine, University of Ottawa, Ottawa, Ontario K1H 8M5, Canada

**Keywords:** Adenovirus, virion stability, viral vectors, helper-dependent Ad

## Abstract

Adenovirus (Ad) vectors are currently the most commonly used platform for therapeutic gene delivery in human gene therapy clinical trials. Although these vectors are effective, many researchers seek to further improve the safety and efficacy of Ad-based vectors through detailed characterization of basic Ad biology relevant to its function as a vector system. Most Ad vectors are deleted of key, or all, viral protein coding sequences, which functions to not only prevent virus replication but also increase the cloning capacity of the vector for foreign DNA. However, radical modifications to the genome size significantly decreases virion stability, suggesting that the virus genome plays a role in maintaining the physical stability of the Ad virion. Indeed, a similar relationship between genome size and virion stability has been noted for many viruses. This review discusses the impact of the genome size on Ad virion stability and emphasizes the need to consider this aspect of virus biology in Ad-based vector design.

## 1. Introduction

Human adenovirus (Ad) is a common pathogen that generally causes minor, self-limiting illnesses in most patients [1]. The basic biology of Ad has been extensively studied for over 60 years, and many aspects of its lifecycle have been elucidated in great detail [2]. This knowledge has allowed for the development of replication-defective Ad vectors that are very efficient at delivering genes to mammalian cells for high-level transgene expression. Indeed, Ad has become the most commonly used vector systems for delivering therapeutic genes in human gene therapy clinical trials [3]. Most Ad vectors are deleted of the essential early region 1 (E1), which renders such vectors incapable of replication in most cell lines [4]. Numerous studies have shown that E1-deleted Ad vectors are ideally suited for studies requiring short-term transgene expression* in vitro* and* in vivo* [4]. Unfortunately, low level viral gene expression from the viral sequences retained in E1-deleted Ad contributes to the immune-mediated loss of the vector and transduced cells* in vivo* [5,6,7], compromising the ability of these vectors to provide long-term transgene expression. Second generation Ad vectors include deletions in the essential E2 or E4 regions, and, in some studies, prolonged transgene expression has been achieved [4]. If a higher cloning capacity is required, fully-deleted or helper-dependent Ad vectors (hdAd) can be generated by removing all viral protein coding sequences [8].

For both E1-deleted Ad and hdAd, removal of some or all viral protein coding sequences is accompanied by an overall reduction in the size of the genome of the virus from 36 kb for the wildtype (AdWT) virus to as small as 30 kb for E1/E3-deleted Ad and, theoretically, ~500 bp for hdAd. These dramatic reductions in the Ad genome length have uncovered a previously unappreciated relationship between vector genome size and virion stability: viruses with genome size below ~90% of the wildtype size have significantly reduced heat stability. Thus, not all Ad vectors are created equal—E1-deleted Ad vectors containing large transgenes, resulting in an overall genome size of greater than ~33 kb, are more stable than vectors containing smaller transgenes. This relationship appears to be a central tenet for several other viruses as well (e.g., bacteriophage lambda, parvovirus). In this review, we discuss our current understanding of the relationship between genome size and virion stability for Ad and other viruses. 

## 2. Adenovirus Biology

Studies of human Adenovirus (Ad) began with its isolation from adenoid tissue and its identification as the cause of some respiratory infections in the early 1950s [9,10]. Human Ad has a remarkable capacity to spread with patients contracting the disease from as few as five virus particles [11]. Ad-induced acute respiratory disease is common in confined populations, such as day care centers, hospitals, retirement homes, and military training venues [12]. Ad accounts for ~8% of all childhood respiratory tract infections, and can lead to bronchitis, bronchiolitis, or pneumonia, requiring hospitalization in ~25% of diagnosed cases [13]. Ads also cause other severe localized diseases, such as colitis, hemorrhagic cystitis, hepatitis, nephritis, encephalitis, myocarditis, and disseminated disease with multiorgan failure [1,14]. Such diseases can be more serious in pediatric and geriatric populations, and in individuals with suppressed immune systems, such as transplant recipients or patients with AIDS.

The discovery that some human Ads are tumorigenic in rodents [15,16] stimulated intensive research into the physiology, genetics, and molecular biology of Ads. These studies, while giving us a great deal of information about DNA replication, control of gene expression, and tumorigenesis [2], also laid the foundations for the later development of Ads as gene transfer vectors. While over 100 serotypes of Ad have been isolated from mammals, birds, reptiles, and amphibians [2], the subgroup C serotypes 2 (Ad2) and 5 (Ad5) have been the most extensively studied. As for many viruses, Ads express viral proteins that alter the cellular environment to promote viral replication. Studies examining Ad infection and lifecycle have uncovered new information regarding host cell DNA replication, mRNA splicing, tumourigenesis, and control of cell cycle progression [17]. For example, alternative splicing, a ubiquitous process in mammalian cells, was first identified in Ad [18,19]. Similarly, association of Ad proteins, such as E1A, with cellular components has led to the discovery or elucidation of the function of these host proteins. One prominent example is the discovery of a 300 kDa protein that binds to E1A: EP300 (E1A-binding protein, 300-kDa), also known as p300 [20]. 

### 2.1. The Ad Genome

**Figure 1:**
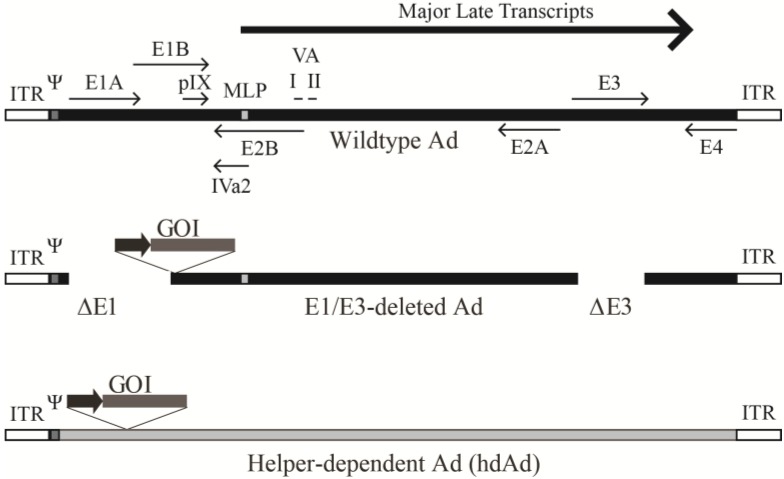
Schematic representation of the adenovirus genome and various adenoviral vectors. (**Top panel**) The Ad5 genome is depicted (not to scale). The early regions are noted as E1–E4 along with the full-length major late transcript from which the L1–L5 transcripts are produced through alternative splicing. Transcripts encoding pIX, IVa2, and VA RNA I and II are also shown. ITR represents the inverted terminal repeats that serve as origins of replication and Ψ is the packaging sequence. The location of the major late promoter is denoted as MLP. Middle panel: A typical early region (E1) deleted Ad vector is shown. The gene of interest (GOI) normally replaces the E1 deletion and its expression is driven by a heterologous promoter (dark arrow). Most of these vectors also have the E3 region removed as it is not essential for viral replication and allows for insertion of a ~8 kb foreign expression cassette. (**Bottom panel**) Helper-dependent Ad schematic. All the DNA encoding viral proteins are removed. Only the ITRs and packaging sequence are required. These vectors usually have non-coding stuffer DNA (grey) to ensure the genome is stable. Adapted from [31].

The genome of Ad2 was the first to be fully sequenced, and it, as well as the Ad5 genome, is approximately 36 kb and encodes over 40 proteins [17]. Adenoviral coding regions are designated early or late depending on when they are expressed (*i.e.*, before or after DNA replication) (Figure 1) [21]. The early regions E1A, E1B, E2, E3, and E4 are the first regions transcribed and encode proteins involved in activating transcription of other viral regions and altering the cellular environment to promote viral production. The E1A proteins induce mitogenic activity in the host cell and stimulate expression of other viral genes [17]. The E2 proteins mediate viral DNA replication, while E3 and E4 proteins alter host immune responses and cell signaling, respectively [22,23]. Activation of the major late promoter (MLP) following the start of virus DNA synthesis allows expression of the late genes encoding primarily virion structural proteins. The late regions (L1–L5) are transcribed from an alternatively spliced transcript. Recently, it was shown that the regions encoding the L4-22K and L4-33K proteins are initially expressed at low levels from a novel promoter located within the L4 region [24], and these proteins act to fully activate the MLP [25]. There are also four small products produced at intermediate/late times of infection, including the structural protein IX (pIX), and the IVa2 protein that helps package viral DNA into immature virions [26]. The late products, VA RNA I and II, inhibit activation of the interferon response, impede cellular micro-RNA processing, and may influence expression of host genes [27,28]. Located on both ends of the genome are the 100 bp inverted terminal repeats (ITRs), which act as the origin of replication, with the ~200 bp viral packaging sequence positioned next to the left ITR. This genome arrangement is common among Ad species [21].

Even though Ad has been studied in great detail for more than half a century, our knowledge of genes encoded by the virus is still expanding. In 2007, Tollefson* et al.* [29] identified a new open reading frame (ORF) located between the fiber ORF and E3, and termed it U exon. The U exon protein (UXP) is expressed from a unique promoter during late stages of infection, and may play a role in virus DNA replication or RNA transcription [25,29]. Similarly, a recent study using deep cDNA sequencing identified many new alternatively spliced transcripts originating from the Ad genome [30], suggesting that there may be numerous other new or altered polypeptides produced by Ad in the infected cell. Thus, the Ad genome may still have many secrets that remain to be uncovered.

### 2.2. The Ad Virion

Ad virions can come in four forms, differing in the quantity of DNA contained in the capsid and whether specific capsid proteins have been processed by the Ad-encoded protease [32,33]. However, only one of these forms, mature virions, are fully infectious. Empty capsids contain no DNA and have the lightest density on a cesium chloride gradient. Light intermediate virions contain only the left end of the Ad genome, whereas heavy intermediates contain the entire genome, but have unprocessed capsid proteins. Mature virions contain the entire genome and have proteolytically processed capsid proteins (IIIa, pVI, pVII, pVIII, pX, pTP, and L1 52/55K). The Ad DNA genome appears to be an important co-factor for conversion of heavy intermediates to mature virions, as treatment of disrupted virions with DNase abolishes protease activity, but this activity can be reactivated through addition of exogenous DNA [34]. This cofactor requirement appears to be simply for a high density negative charge, rather than a specific need for Ad DNA, since any DNA, or even ssDNA or poly-glutamic acid, can function as a cofactor. Thus, the Ad DNA plays an active role in maturation of the Ad virion.

**Figure 2:**
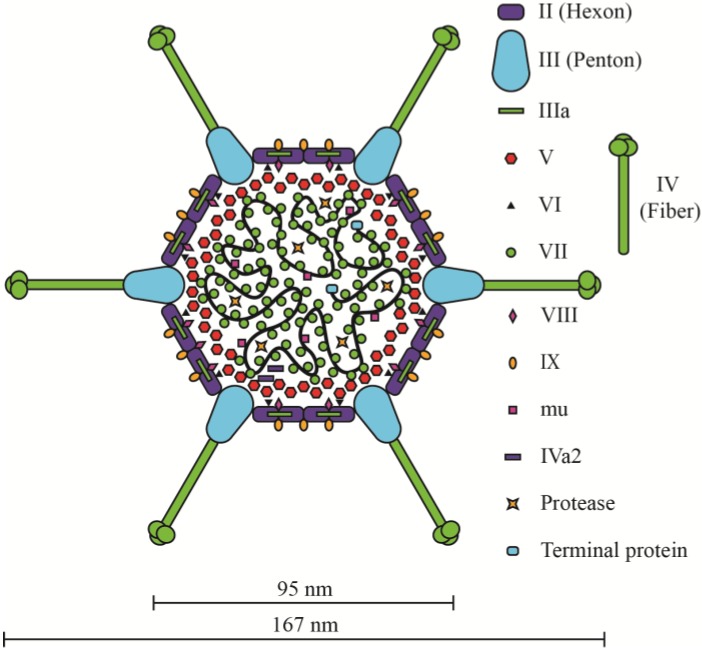
Model of the Ad5 virion, based on data provided in [26,35,36,37,38]. Adapted from [79].

The general structure of the mature Ad virion is conserved among different Ad species. The main “body” of the virion consists of a non-enveloped icosahedral capsid with a diameter of ~70–100 nm surrounding the DNA-protein core (Figure 2). The fiber protein that extends from each vertex of the icosahedron can increase the overall diameter of the virus by more than two-fold. The Ad5 capsid is composed of three major proteins (II, III, and IV) and five minor proteins (Figure 2) [26,35,36,37,38]. Trimers of polypeptide II, called hexons, form the triangular facets of the capsid and contribute ~80% of the capsid mass [39]. Pentons are pentamers of polypeptide III that cap the vertices and serve as the base from which fiber (trimers of protein IV) extend outwards. The five minor polypeptides (IIIa, IVa2, VI, VIII, and IX) act to stabilize the capsid or connect the outer capsid to the nucleoprotein core [39]. Within the virion, the viral DNA is bound to the basic proteins VII, V, and Mu [40,41,42]. Protamine-like protein VII condenses and compacts the DNA, forming a central dense core and facilitates DNA packaging within the physical constraints of the capsid [43]. Parts of the protein-wrapped DNA form spherical structures, termed adenosomes, that are pushed into the vertex regions of the inner capsid [44]. Protein V in believed to form a shell surrounding the protein VII-DNA complex and serves as a key linkage protein between the DNA core and inner capsid [44,45,46,47]. Protein V is reported to interact directly with penton protein [48,49]. Protein V also interacts indirectly with penton and peripentonal hexon through protein IIIa, and again indirectly with the remainder of hexon through protein VI [36,37,48,50,51,52]. A very recent refinement of the position of some the minor Ad capsid proteins has suggested that protein V interacts with both protein VI and VIII on the interior of the capsid (underneath the vertex regions), and that protein IIIa is located largely on the exterior of the capsid [38]. Interestingly, protein V is only found in the genus Mastadenoviruses (which includes human, simian, porcine and canine adenoviruses, amongst others), but not in Aviadenovirus, Atadenovirus and Siadenovirus [21]. Pre-mu is a 79 amino acid protein that may also be involved in compacting the viral DNA [53]. Cleavage of pre-mu by the Ad protease is speculated to relax the viral DNA in preparation for delivery to the nucleus [53,54]. Overall, the nucleoprotein core does not have an ordered structure that is visible in cryo-EM images [55], unlike bacteriophage T4 and lambda or Herpes virus [55,56,57], which has prevented elucidation of its true structure and conclusive identification of all points of contact between the core and inner capsid.

### 2.3. Packaging of Ad DNA into the Capsid

The process and proteins involved in mediating Ad DNA packaging into the capsid are beginning to be elucidated. As mentioned, the packaging sequence contained in the Ad DNA is approximately 200 bp and is located immediately adjacent to the left inverted terminal repeat (ITR) and before the E1 coding sequence [58]. Its placement at the left end of the genome leads to Ad DNA being encapsidated in a polar fashion, and virions that have packaged only subgenomic DNA fragments only contain left-end sequences [59]. However, the packaging sequence can function equally well when transposed to the right end of the Ad genome [58], and, for such a virus, the polar packaging is reversed. Ad vectors have been created that contain packaging sequences at both ends of the genome [60,61,62], which arguably may enhance the efficiency of vector DNA packaging, as it can initiate at either end. The packaging sequence becomes non-functional when moved more than ~300 bp internal from the end of the genome [58].

The packaging sequence contains seven functionally redundant elements referred to as A repeats (TTTGN8CG) [63,64]. The virus-encoded IVa2 protein binds the CG motif of the A repeat either as a homodimer, or in complex with the L4 22K protein, which itself binds the TTTG motif in the A repeat [65,66]. L4 22K must form a complex with IVa2 in order to successfully bind the packaging sequence [67,68]. The L4 33K protein, which shares amino-terminal homology with the L4 22 K protein, has also been found bound to the DNA packaging sequence, but again only in the presence of IVa2 [69]. Other proteins involved in the DNA packaging process include L1-52/55K [70,71,72] and IIIa [73]. Loss or mutation of any of these proteins results in an inability to form mature virions. However, such viruses are still capable of forming empty capsids which lack any viral DNA, suggesting that capsids are preformed and do not “nucleate” on the packaging sequence [70,71]. Indeed several groups have shown that the packaging sequence can be entirely deleted from the Ad DNA and significant amounts of empty capsid are still formed [74,75,76]. Thus, it is believed that the Ad capsid forms first within the nucleus and the Ad DNA is subsequently spooled into the preformed capsids [77]. IVa2 may be part of a portal structure, and act as the ATPase-dependent motor that facilitates spooling of the Ad DNA into the preformed capsids [26,78]. Consistent with this idea, the IVa2 protein is found at only one vertex within the mature Ad virion [26].

### 2.4. Adenovirus Infection

Ad5 entry into host cells is initiated by the binding of fiber protein to the coxsackie and adenovirus receptor (CAR), which is expressed on the surface of many cell types [80,81]. Ad5 can also use heparin sulfate glycosaminoglycans (HSGs) on the cell surface for infection [82]. This process can be achieved through either direct binding of fiber to HSG or through a bridging interaction mediated by binding of Ad hexon to various blood factors or the complement component C4-binding protein [83,84,85]. An arginine-glycine-aspartic acid (RGD) motif in penton protein then interacts with membrane α_v_β_3_ or α_v_β_5_ integrins to induce Ad internalization via receptor-mediated endocytosis into clathrin-coated vesicles, which eventually form endosomes [86]. The virus evades degradation by lysing the endosomal membrane and escaping from the early endosome [87,88]. The virion then migrates to the nucleus by travelling along microtubules [87], and the capsid proteins are dismantled in a step-wise manner during transport [89]. The capsid proteins are degraded in the cytoplasm while the protein VII-viral DNA complex makes its way into the nucleus through the nuclear pore [42,89,90]. Entry into the nucleus is followed by early gene expression, viral DNA replication, late gene expression, and assembly of progeny virions [2]. During at least the early stage of viral gene expression, AdWT (and Ad vector) DNA uses cellular histones to form DNA structures similar to cellular chromatin, which appears necessary for efficient viral gene expression [31,79,91,92,93].

## 3. The Ad Genome Size and Virion Stability

### 3.1. Early Region 1-Deleted Ad Vectors

Ad-based vectors are one of the most commonly used vehicles for delivery of transgenes into mammalian cells in research laboratories worldwide, and in human gene therapy clinical trials [3]. As such, several studies have explored their cloning capacity for foreign DNA and how this impacts upon the function and stability of the vector. Although most of the following information is gleaned from studies of human Ad serotype C, given the overall similarity between human and other Ads, it is likely (but not certain) that these principles hold true to all Ads in general. Studies have shown that the Ad capsid can accommodate up to ~108% of the wildtype genome length [94,95]. However, such vectors tend to be unstable and undergo spontaneous genome rearrangement, usually resulting in a loss of the encoded transgene and a reduction in the overall genome size to closer to wildtype length [94]. Vectors with an overall genome size of ~105% of the wildtype genome length tend to be stable, resulting in a functional cloning capacity of ~8 kb in E1/E3-deleted Ad vectors [94] (Figure 2). On the other end of the spectrum, E1/E3-deleted Ads encoding expression cassettes for a fluorescent protein or short-hairpin RNA may have a genome size much smaller than wildtype Ad, in the range of 30–32 kb, and these genomes are relatively stable (*i.e.*, no evident selection for rearrangement) [96,97]. Thus, E1/E3-deleted Ad vectors can vary greatly in their overall genome size from as small as 30 kb to over 38 kb. These E1-deleted vectors are grown in E1-complementing cell lines, such as the extensively used 293 cell line [98].

### 3.2. Helper-Dependent Ad Vectors

The latest generation of Ad vectors, hdAds, do not encode any viral proteins and only need the packaging signal for DNA packaging into the capsid and the ITRs to allow for genome replication [99] (Figure 2). Thus, hdAd vectors allow for almost 38 kb in cloning capacity. Since hdAd are devoid of most if not all viral genes, they lack the ability to replicate autonomously and typically rely on an accompanying “helper” virus to provide replicative and packaging functions *in trans*. Early systems used to generate hdAd vectors relied on either wildtype or E1-deleted helper viruses to provide these necessary functions [100,101,102,103,104,105]. As cesium chloride density gradient-based purification separates virions based on the genome length (which directly affects the density of the virion [61]), the hdAd could be partially purified away from the helper virus by centrifugation. Interestingly, hdAd with very small genome sizes tended to undergo spontaneous rearrangement to increase the length of the genome to closer to wildtype size [101,102], an early suggestion that there was an optimal genome size for Ad DNA packaging. 

An alternative system for generating Ad vectors deleted of most viral protein coding sequences involved the use of an E1-deleted Ad containing one loxP site, a target for the bacteriophage P1 Cre recombinase, located immediately down-stream from the transgene cassette in the E1 region and a second site inserted within the E3 region [106]. In the presence of Cre, the loxP sites were recombined, excising the Ad coding sequences located between the two loxP sites, and leaving a genome of approximately 9 kb. The E4 coding region was retained in these vectors. In this system, the unrecombined vector itself acts as the complementing helper. Interestingly, the protein content of the virions containing the 9 kb genome was quite aberrant, with many extra protein bands which were postulated to be nonspecific degradation products.

The current system for generating hdAd involves the use of an E1/E3-deleted helper virus that contains a packaging sequence flanked by Cre or Flp recombinase recognition sites and a 293-based cell line that expresses the corresponding recombinase [99,107,108,109,110]. This combination results in a helper virus that can replicate and provide all the trans-acting functions necessary for replication and packaging of the hdAd, but the helper virus is deficient in its ability to package its own DNA due to Cre- or Flp-mediated removal of the viral packaging sequence. Additional improvements to the original Cre/loxP system for hdAd preparation have involved modifying the packaging sequence to reduce the chances of recombination between the helper virus and the hdAd genomes, which could result in loss of one of the loxP sites and outgrowth of the modified helper virus [111,112]. 

Early during the development and characterization of hdAd, we, and others, showed that hdAd vectors with genomes below ~27 kb in size spontaneously rearrange their DNA to increase the genome size [61,101,102]. Indeed, this property can be used to design hdAd vectors containing two (or more) copies of a desired transgene, as a vector constructed to be 15 kb in size will be packaged as a mix of direct and inverted dimers, effectively giving a vector genome size of 30 kb [61,62,113]. However, most hdAd are constructed of a size greater than at least 27 kb to promote stable genome structure, frequently through the inclusion of non-coding “stuffer” DNA [114,115]. For example, a 5 kb expression cassette would require ~22 kb of stuffer DNA to reach the 27 kb genome size minimal limit. Importantly, the nature of the stuffer DNA can significantly affect vector function. An hdAd containing 22 kb of eukaryotic stuffer DNA expressed its transgene ~10-fold higher* in vitro* and* in vivo* compared to a vector with an identical expression cassette but containing stuffer DNA derived from prokaryotic DNA [114]. Similar results have been observed by others [116]. Subsequent studies showed that the prokaryotic stuffer DNA was subjected to epigenetic regulation which down-regulated expression of the associated transgene [115]. Nevertheless, when constructed optimally, hdAd are associated with a high transduction efficiency and low toxicity, and can provide long-term and high levels of transgene expression in a variety of species [4,8,117,118].

### 3.3. Virion Instability of Ad Vectors

**Figure 3:**
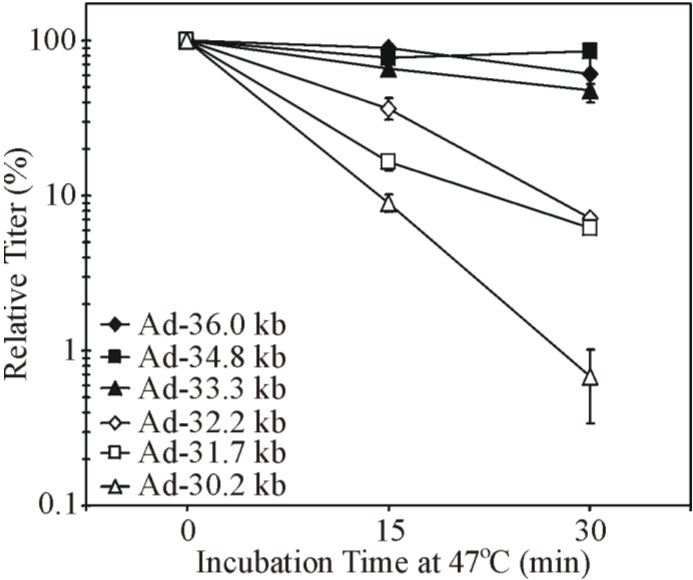
Ad genome size affects Ad heat stability. E1/E3-deleted Ad vectors with genome size ranging from 30.2 to 34.8 kb and wildtype Ad (AdWT) were heated at 47 °C over a timecourse of 30 min. The viral titer was determined at each timepoint to assess virion stability. Viruses with genomes over ~33.3 kb were as stable as the 36 kb AdWT. Viruses with smaller genomes exhibited increased instability as genome sizes decreased. Adapted from [125,126].

In an attempt to devise a new method for hdAd production that results in vector of greater purity, we designed a novel approach which takes advantage of the DNA packaging restrictions imposed on Ad capsids deficient in protein IX (pIX) [119]. pIX is a minor capsid protein that assists in holding the facets of the virion together. Capsids deficient in pIX are believed to accommodate a maximum of 35 kb of DNA, and thus cannot package a full-sized AdWT genome [120,121]. pIX-deficient virions are also heat labile [122]. We hypothesized that a helper virus with a genome greater than 35 kb could be grown in 293 cells expressing pIX while in normal 293 cells, which encode but do not naturally express pIX [123,124], the helper virus would be unable to produce viable virions due to the inability to package its own large genome into pIX-deficient capsids. As a result, hdAd with a genome smaller than 35 kb (e.g., 29.6 kb [119]) would be selectively packaged during hdAd propagation. This new system functioned largely as expected [119]. Although the hdAd generated with this system were heat labile, as expected since the virions were deficient in pIX, this effect appeared to be independent of pIX, as hdAd virions with an identical DNA genome and containing pIX in the virion showed similar heat sensitivity [125]. Interestingly, heat stability could be rescued simply by increasing the size of the hdAd genome from 29.6 kb to ~34 kb. Similar results were obtained when E1/E3-deleted Ad vectors were used. E1/E3-deleted Ad with a genome larger than ~33 kb were just as stable as AdWT while those smaller than ~32 kb showed increased instability as genomes decreased in size (Figure 3) [125]. While capsid morphology and protein content did not differ between the various Ad vectors, the capsids of Ads that had smaller genomes quickly fell apart when heated [125]. Specifically, heating resulted in a release of the vertex proteins (fiber, penton, and, likely, the peripentonal hexon), followed by complete disintegration of the capsid structure. This suggests that the biophysical connections, which normally hold these proteins in place, are compromised when the genome is reduced in size. Thus, the Ad genome size contributes to the physical stability of Ad. 

The mechanism by which Ad DNA contributes to virion stability is not yet fully elucidated. In the virion, the nucleoprotein core extends into the vertex regions of the inner capsid, and makes direct contact with penton and peripentonal hexon. This region is destabilized during virus endocytosis at the early stages of infection. Binding of Ad penton to membrane integrins may cause conformational changes in the peripentonal capsid region [89], resulting in liberation of protein VI which functions to lyse the endosomal membrane [88]. Since timely removal of the vertex proteins is crucial for efficient infection, this process may be highly regulated and require proper positioning of all viral proteins involved. Packaging of the tightly condensed full-length genome into the capsid may position the DNA correctly within the vertex regions to allow the proper protein chain of connections to stabilize the vertex proteins. Reducing the size of the genome may affect correct positioning and prevent the interaction of these proteins, ultimately destabilizing the capsid.

The mechanism of virion instability may also vary depending on the size of the DNA. Virions with very small genomes (~9 kb) show aberrant protein content [106,127], suggesting that the small genome may affect proper maturation of the virion. As the Ad DNA acts as a cofactor for activation of the Ad protease [34], it is possible that the small genome is not capable of efficiently activating the protease, or loose packaging of the viral DNA may affect the ability of an active protease to interact with its substrate. For vectors with genomes of ~29 kb, there was no obvious defect in protein content [125], suggesting the protease was capable of efficiently mediating virion maturation. A recent study by Benevento* et al.* [128] used quantitative proteomics to characterize the protein composition of Ad virus, and also the effect of thermal denaturation on virion disassembly. It is likely that a similar approach could be used to delve into the mechanism by which the Ad genome affects virion stability.

## 4. Studies of Genome Size and Virion Stability in Other Viruses

The relationship between genome size and capsid stability has been studied in other viruses as well. For bacteriophage lambda, another icosahedral virus, genomes smaller than 78% of the wildtype length show reduced infectivity while genomes larger than 106% are not packaged [129]. The tight packaging of bacteriophage DNA into the capsid causes significant electrostatic repulsion, which results in an increase in internal pressure [129,130,131]. This internal pressure is not only used to inject the phage DNA into bacteria during the infection process, but also directly influences capsid strength and stability. A previous study by Ivanovska* et al.* [129] observed that phages with smaller genomes were half as strong as those with full length genomes, as measured by atomic force microscopy. Thus, the pressure exerted by the packaged DNA makes the capsid physically stronger. In terms of Ad, increased capsid pressure due to electrostatic interactions are unlikely to play a role in capsid stability since the basic proteins VII, V, and µ likely function to neutralize these charges in the packaged DNA. However, atomic force microscopy measurements of the “strength” of Ad virions containing varying lengths of DNA could be very informative [132,133].

Studies with other viruses, such as the single-stranded DNA (ssDNA) mouse parvovirus minute virus (MVM), also show that the genome is important in capsid stability. When the capsid strength of empty and wildtype MVM capsids were compared, those containing DNA were stronger by 140% [134]. MVM DNA binds to 60 concavities of the inner capsid and interacts with the VP2 capsid protein to increase capsid thickness, strength and stability [134]. Mutation of key amino acid residues that mediate the DNA-VP2 interaction reduced virus infectivity, heat stability, and virion rigidity [135,136]. Similar observations and mechanism have been proposed for genome-mediated capsid stabilization of the cowpea chlorotic mottle virus (CCMV) and bean pod mottle virus (BPMV) [137,138,139]. 

An inverse relationship between genome size and capsid stability was recently observed in adeno-associated viruses (AAVs). AAVs are small, non-enveloped, ssDNA viruses that also belong to the *Parvoviridae* family. Horowitz* et al.* studied the effects of increasing heat on AAV capsids containing genomes varying from 72% to 123% of the wild-type length [140]. AAVs with smaller genomes required higher temperatures to induce capsid dissociation compared to wildtype AAV. These researchers suggested that packaging of sub-genomic DNA resulted in reduced internal pressure, which lessened the outward-directed pressures on the capsid, essentially increasing the stability. Several researchers have examined the ability of AAV capsids to accommodate large genomes, after an initial report suggested that very large ssDNA genomes could be found in virions (~180% of the wildtype genome length [141]). These three follow-up studies clearly showed that AAV has an upper limit of ssDNA packaging of ~5.2. kb, and that attempts to package larger genomes simply resulted in the packaging of sub-genomic fragments, which was associated with a dramatic decrease in vector recovery [142,143,144]. Apparent packaging of larger genomes was likely the result of recombination between these subgenomic fragments in transduced cells, resulting in restoration of the intact large genome. The observation that attempts to package very large genomes is accompanied by a significant drop in virus recovery once again illustrates the importance of optimal genome size in vector design.

Thus, many icosahedral viruses have a very strong preference for genome size, with an associated disruption in virus stability and viability when this preferred size is dramatically altered. The mechanism by which the nucleic acid contributes to capsid stability likely depends on the virus and the nature of genetic material (*i.e.*, dsDNA, ssDNA, or RNA).

## 5. Implications for Vector Design

The relationship between the genome size and virion stability is important to consider when designing and cloning novel vectors. As shown in Figure 3 [125], a ~32 kb Ad vector is less stable than a ~35 kb vector. However, how this innate instability impacts vector function is not completely clear. Ad vectors deficient in pIX have a similar virion instability as vectors with small genomes, and pIX-deficient vectors reportedly have an enhanced ability to activate peripheral blood mononuclear cells [145]. Thus, unstable Ad vectors may have a greater tendency to elicit anti-vector immune responses. If capsids of Ad vectors with small genomes undergo significant disassembly in the early endosome, due to this inherent instability, the vector DNA may be exposed to pathogen recognition receptors, such as toll-like receptor 9 (TLR9), which can recognize the high CpG content of Ad DNA and initiate innate immune response signaling cascades [146,147]. Moreover, if vectors with small genomes have a slight defect in their ability to escape the early endosome, due to inappropriate release of the endosomal disrupting protein VI [88], enhanced innate immune activation can also occur due to transit of the vector into the late endosome [148,149]. A similar phenomenon could occur for AAV vectors with small genomes, as their enhanced stability could alter their intracellular trafficking and engagement of cellular immune pattern recognition receptors. Indeed, it has been suggested that non-coding “stuffer” DNA could be incorporated into the AAV vector DNA to increase the size of the genome closer to wildtype size to preserve natural vector stability and function [140]. As such, the overall genome size is important to consider in the design of vectors derived from a number of different viruses.

## 6. Conclusions

Viruses have been under selective pressure for millions of years, and have optimized the design of their genome to promote rapid and efficient expression of viral genes within the infected host cell. It is clear that these same evolutionary pressures have optimized the relationship between the genome size and the capsid that is responsible for delivering the viral genetic material to the host. For adenovirus, we, and others, have shown that genomes below 75% or above 105% of the wildtype genome length tend to rearrange to achieve a size closer to wildtype length. However, although genome stability can be achieved at 75% of the wildtype genome length [61], virion stability is only achieved with a genome size of ~90% of the wildtype genome length. Many other viruses show a similar dependence between genome size and capsid stability, suggesting this is a general property for many viruses. 
